# Propranolol attenuates hemorrhage and accelerates wound healing in severely burned adults

**DOI:** 10.1186/s13054-015-0913-x

**Published:** 2015-05-04

**Authors:** Arham Ali, David N Herndon, Ashish Mamachen, Samir Hasan, Clark R Andersen, Ro-Jon Grogans, Jordan L Brewer, Jong O Lee, Jamie Heffernan, Oscar E Suman, Celeste C Finnerty

**Affiliations:** Department of Surgery, University of Texas Medical Branch, 301 University Boulevard, Galveston, TX 77555 USA; Shriners Hospitals for Children, 815 Market Street, Galveston, TX 77550 USA; School of Medicine, University of Texas Medical Branch, 301 University Boulevard, Galveston, TX 77555 USA; Institute for Translational Sciences and the Sealy Center for Molecular Medicine, University of Texas Medical Branch, 301 University Boulevard, Galveston, TX 77555 USA

## Abstract

**Introduction:**

Propranolol, a nonselective β-blocker, exerts an indirect effect on the vasculature by leaving α-adrenergic receptors unopposed, resulting in peripheral vasoconstriction. We have previously shown that propranolol diminishes peripheral blood following burn injury by increasing vascular resistance. The purpose of this study was to investigate whether wound healing and perioperative hemodynamics are affected by propranolol administration in severely burned adults.

**Methods:**

Sixty-nine adult patients with burns covering ≥30% of the total body surface area (TBSA) were enrolled in this IRB-approved study. Patients received standard burn care with (n = 35) or without (control, n = 34) propranolol. Propranolol was administered within 48 hours of burns and given throughout hospital discharge to decrease heart rate by approximately 20% from admission levels. Wound healing was determined by comparing the time between grafting procedures. Blood loss was determined by comparing pre- and postoperative hematocrit while factoring in operative graft area. Data were collected between first admission and first discharge.

**Results:**

Demographics, burn size, and mortality were comparable in the control and propranolol groups. Patients in the propranolol group received an average propranolol dose of 3.3 ± 3.0 mg/kg/day. Daily average heart rate over the first 30 days was significantly lower in the propranolol group (*P* <0.05). The average number of days between skin grafting procedures was also lower in propranolol patients (10 ± 5 days) than in control patients (17 ± 12 days; *P* = 0.02), indicative of a faster donor site healing time in the propranolol group. Packed red blood cell infusion was similar between groups (control 5.3 ± 5.4 units vs. propranolol 4.4 ± 3.1 units, *P* = 0.89). Propranolol was associated with a 5 to 7% improvement in perioperative hematocrit during grafting procedures of 4,000 to 16,000 cm^2^ compared to control (*P* = 0.002).

**Conclusions:**

Administration of propranolol during the acute hospitalization period diminishes blood loss during skin grafting procedures and markedly improves wound healing in severely burned adults. As burn patients require serial surgical interventions for motor and cosmetic repair, restricting blood loss during operative intervention is optimal.

## Introduction

A severe burn injury is characterized by a profound increase in metabolism, far beyond that produced by other forms of trauma. Hypermetabolism is mediated by a surge in stress hormones including catecholamines [1,2] and glucocorticoids and may persist long after the initial burn insult [3,4]. Cardiac stress following burn injury is characterized by increased cardiac work, cardiac output, resting heart rate, rate pressure product, and stroke volume. If left untreated, these perturbations in cardiac physiology contribute greatly toward postburn morbidity and mortality.

Hyperdynamic changes to the cardiovascular system are frequently associated with copious amounts of operative blood loss. Paired with inadequate resuscitative efforts and shifts in fluid compartments, these changes cause many patients with burn injury to become hemodynamically unstable. Additionally, as patients with burn injury undergo serial skin grafting procedures, limiting hemorrhage during these operations becomes paramount to early recovery.

**Figure 1:**
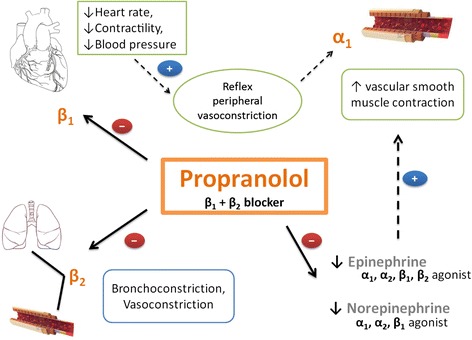
Proposed mechanism by which propranolol induces peripheral vasoconstriction. Induction of peripheral vasoconstriction by propranolol can be attributed to three main actions. (1) Inhibition of β_1_ receptors in the heart decreases cardiac output, thereby inducing reflexive peripheral vasoconstriction via stimulation of α_1_ receptors in vascular smooth muscle. (2) Direct inhibition of β_2_ receptors incites peripheral vasoconstriction. (3) By blocking β-adrenergic effects of circulating catecholamines epinephrine and norepinephrine, α_1_-adrenergic receptor effects remain unopposed, resulting in vascular smooth muscle contraction. Solid arrows indicate direct effects, and broken arrows indicate indirect effects.

Propranolol, a nonselective β-blocker, has widespread systemic effects following burn injury. Recently, we reported that administration of propranolol in children with severe burn injury for one year significantly improves body composition, resting energy expenditure, and cardiac function [5]. Attenuation of cardiac sequelae occurred in a dose-dependent manner with the most favorable results noted at a dose of 4 mg/kg/day [6]. Peripherally, propranolol decreases lower limb blood flow by increasing leg vascular resistance in severely burned adults [7]. Induction of peripheral vasoconstriction by propranolol has led to the successful implementation of the β-blocker in the management of infantile hemangiomas, [8,9] variceal bleeding, [10] and recurrent epistaxis [11]. An overview of the mechanisms by which propranolol induces peripheral vasoconstriction is presented in Figure 1. Whether or not the effects of these changes on cardiovascular function alter operative hemorrhage or wound healing times remains to be determined. Here we report the perioperative effects of propranolol administration in adults with severe burn injury in a prospective, single-institution study.

## Materials and methods

### Patient enrollment and stratification

**Figure 2:**
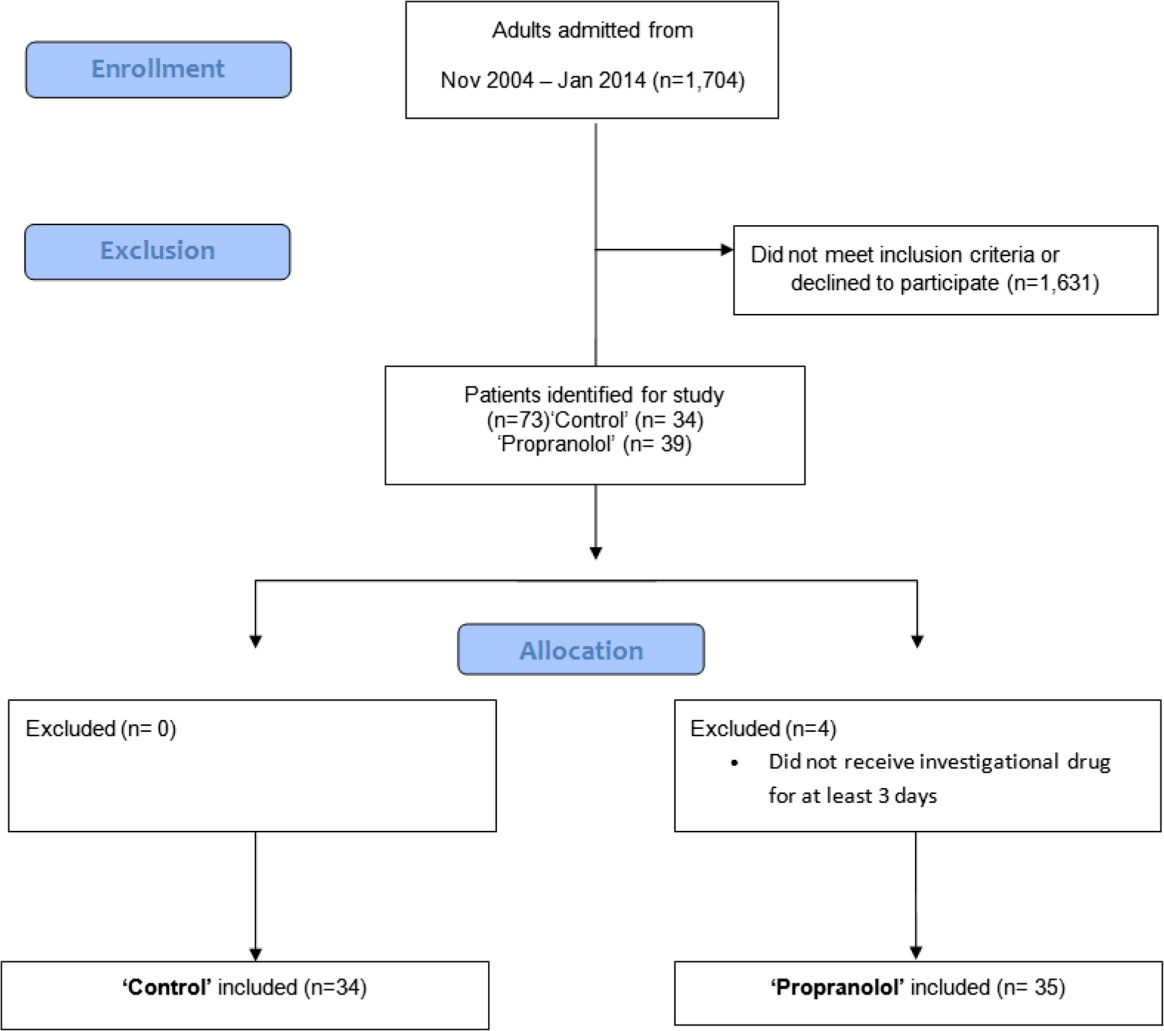
Patient enrollment diagram.

One thousand seven hundred four patients were admitted or referred to the Blocker Burn Unit at the University of Texas Medical Branch between November 2004 and 1 January 2014. One thousand six hundred thirty-one patients did not meet the following inclusion criteria: older than 18 years, burn wounds covering ≥30% of the total body surface area (TBSA), treatment with at least one surgical skin grafting procedure, and consent to participate in data collection (Figure 2). All patients meeting the inclusion criteria provided freely tendered consent to participate in the study, which was approved by the Institutional Review Board of the University of Texas Medical Branch (Galveston, TX). The treatment protocols were stable during the study period, as determined by stability in the length of hospital stay (LOS) (0.5 days per percent TBSA burned), morbidity (infections, lung injury, and acute kidney injury), and mortality. Four patients randomized to the propranolol group never received propranolol and were excluded from the study. The data presented here were collected from 69 adult patients with burns who received standard of care established at our hospital and described in detail previously [12,13]. Within 48 hours of hospital admission, patients received either standard burn care treatment (control; n = 34) or standard burn care treatment plus propranolol (propranolol; n = 35), which was administered throughout hospitalization to decrease baseline heart rates by approximately 20%. Target dosing was achieved as previously described [5]. If bradycardia occurred, the following dose of propranolol was withheld. Propranolol administration was re-initiated after 16 hours beginning at one half of the original dose. Thereafter, the dose was titrated back to target levels over the following 48 hours.

### Nutritional support and wound care

**Figure 3:**
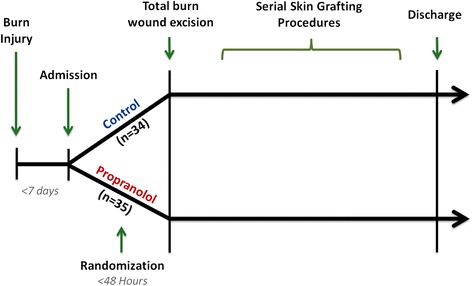
Patient enrollment and timeline of hospital course. Patients were admitted within 7 days of burn injury. Over the next 48 hours, patients were randomized to control (n = 34) or propranolol (n = 35) groups and then underwent total burn wound excision. Thereafter, patients underwent serial skin grafting procedures once donor sites wounds healed. Patients were then discharged once wounds were deemed to be 95% healed.

Over the first 24 hours following admission, resuscitation was accomplished per the Parkland formula (4 ml × weight (kg) × TBSA burned (%)) with lactated Ringer’s solution given incrementally [14]. Hypovolemia was treated with crystalloid or colloid fluids based on the individual clinician’s preference. Vasotropes or inotropes were used when patients did not respond to volume infusion or if the patient was in septic shock. Total burn wound excision was performed on all patients within 48 hours of admission. Autograft and homograft skin were used to cover the wounds. Grafting procedures were repeated once the donor site wounds healed (approximately once a week). Therefore, the time in between grafting procedures indicated approximate wound healing times. Patients were discharged once wounds were deemed to be 95% healed. An outline of the course of surgical interventions from the time of burn injury to the time of discharge is provided in Figure 3.

Nutrition was provided by continuous nasoduodenal tube feeds in the form of Vivonex total enteral nutrition (composition: 82% carbohydrate, 15% protein, and 6% fat) (Nestle HealthCare Nutrition, Inc., MN, USA). Nutrition and metabolism were monitored by assessing serum levels of albumin, transthyretin, and retinol-binding protein. Weight and urinary output were measured daily.

### Perioperative blood loss

Blood loss was estimated visually by the surgical and anesthesiology teams as described in surgical literature [15,16]. Perioperative hematocrit levels were defined as values obtained within 12 hours before or after skin grafting procedures. If multiple values were recorded, hematocrit values obtained closest to the time of operative intervention were used. Patients lacking corresponding hematocrit values within this time frame were omitted.

### Statistical methods

Data with normal distribution were analyzed using an unpaired Student’s *t* test and Fisher’s exact test. Data with unequal or skewed distribution were either transformed or analyzed using Mann-Whitney rank-sum tests. Patient demographic and burn injury characteristic data were described as mean ± standard deviation or counts (percentages) where appropriate. Propranolol dosing, daily heart rates, wound healing, skin grafting procedure, and blood loss data were described as mean ± standard deviation unless otherwise noted.

The relationship between change in preoperative and postoperative hematocrit levels and skin graft area was modeled by linear regression to a quadratic curve and included an interaction between treatment group (control vs. propranolol) and skin graft area. Skin graft area was log transformed for improved centering. A likelihood ratio test was used to assess the significance of treatment effect. Change in hematocrit vs. skin graft area was described as adjusted means ± 95% confidence intervals. Statistical analysis was performed using R statistical software (R Core Team, 2013, version 3.1.1). A 95% level of confidence was assumed.

## Results

### Patient disposition and burn injury characteristics

Sixty-nine patients with ≥30% TBSA burns were evaluated; 34 adult burn patients were randomized to the control cohort, and 35 adult burn patients were randomized to the propranolol cohort. Age, sex, etiology of burn injury, burn to admission time, and duration of acute hospitalization were similar between cohorts (Table 1). Although TBSA burned was higher in control patients than in propranolol patients (59 ± 22% vs. 49 ± 18%; *P* = 0.04), the percent of full-thickness burns was similar between groups (control 45 ± 29% vs. propranolol 40 ± 22%; *P* = 0.48), indicating similar burn severity.

**Table 1:** **Patient demographics and burn injury characteristics**

**Parameter**	**Control (N = 34)**	**Propranolol (N = 35)**	***P*** **value**
**Age, yr**	38 ± 16	41 ± 14	0.33
**Sex, males (%)**	30 (88)	29 (83)	0.73
**Burn type, n (%)**			
Electrical	1 (3)	2 (6)	0.96
Flame	30 (88)	30 (86)	
Other	3 (9)	3 (8)	
**TBSA burn,%**	59 ± 22	49 ± 18	0.04
**TBSA third,%**	45 ± 29	40 ± 22	0.48
**Burn to admission, d**	1 ± 1	1 ± 3	0.36
**LOS (survivors), d**	52 ± 54	46 ± 35	0.99
**LOS (nonsurvivors), d**	30 ± 34	20 ± 9	0.50
**LOS/TBSA, d**	0.8 ± 0.8	0.9 ± 0.6	0.30
**Mortality, n (%)**	10 (29)	6 (17)	0.36

Data are presented as mean ± standard deviation or count (percentage). LOS, length of stay; TBSA, total body surface area.

### Propranolol dosing

Patients in the propranolol group received an average dose of 3.3 ± 3.0 mg/kg/day for an average of 40 ± 40 days. Propranolol was initiated on average, 3 ± 3 days postburn.

### Cardiac function

**Figure 4:**
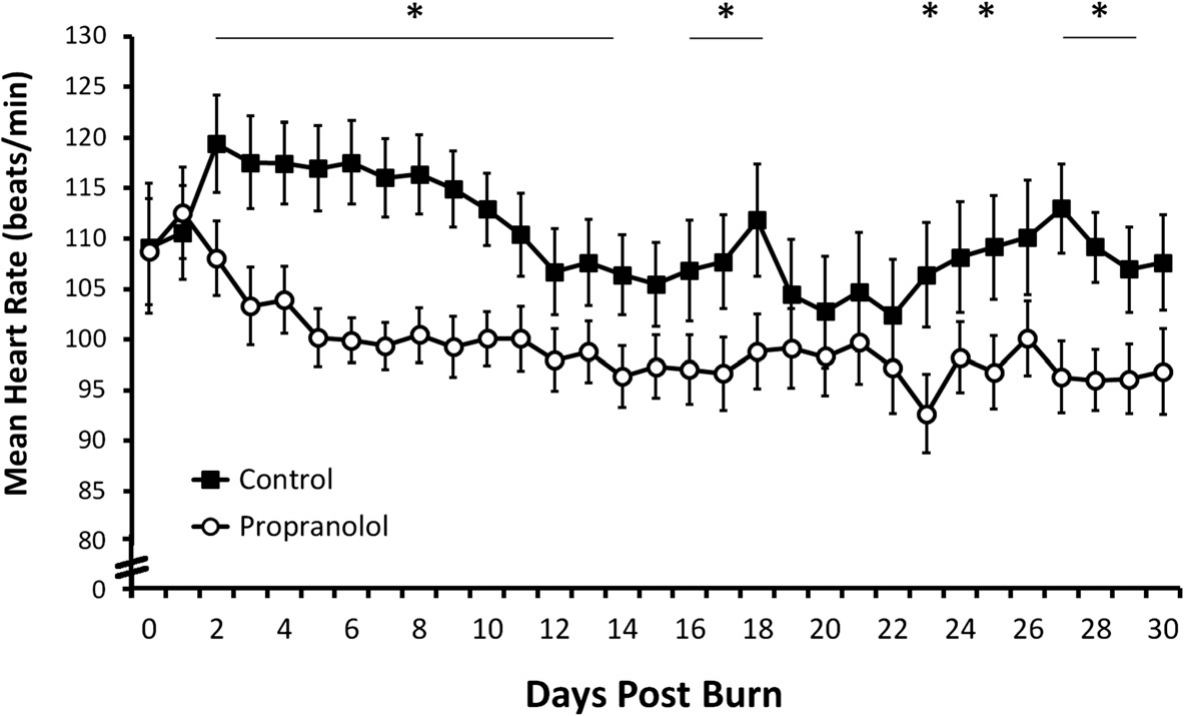
Daily heart rate. Daily mean heart rate was significantly lower in patients on propranolol than in control patients. Data are presented as mean ± standard error of the mean. ^*^ *P* <0.05.

Daily mean heart rate was significantly decreased in patients receiving propranolol compared to control (Figure 4; *P* <0.05). Heart rates between postburn days 2 to 30 in propranolol patients were, on average, 11 ± 4 beats per minute lower than those in the control group. Control patients remained tachycardic (>100 beats/min) throughout hospitalization. Conversely, normal heart rates were achieved in the propranolol group by postburn day 7, with a relatively sustained decrease in heart rate by postburn day 12.

### Wound healing

The average number of skin grafting procedures each patient underwent was the same for each group (4 ± 3 procedures; *P* = 0.90; Table 2). However, the time between skin grafting procedures was lower in the propranolol cohort than in the control cohort (10 ± 5 days vs. 17 ± 12 days; *P* = 0.02). These data indicate that propranolol significantly improves donor site wound healing time by an average time of one week compared to the control treatment. Graft type was not different between groups (data not shown).

**Table 2:** **Wound healing and skin grafting procedures**

**Parameter**	**Control**	**Propranolol**	***p*** **Value**
**Wound healing**			
**Number of SGP/patient**	4 ± 3	4 ± 3	0.90
**Duration between SGP (days)**	17 ± 12	10 ± 5	**0.02**
**Skin grafting procedures**			
**Skin graft area (cm** ^**2**^ **)**	3,300 ± 4,800	4,500 ± 4,000	**0.01**
**Preoperative HCT (%)**	32 ± 9	30 ± 8	0.05
**Postoperative HCT (%)**	28 ± 6	28 ± 5	0.24
**PRBC transfused (units)**	5.3 ± 5.4	4.4 ± 3.1	0.89
**EBL/graft area (ml/cm** ^**2**^ **)**	0.37 ± 0.73	0.26 ± 0.21	0.70

Data presented as mean ± standard deviation. EBL, estimated blood loss; HCT, hematocrit; PRBC, packed red blood cells; SGP, skin grafting procedures.

### Blood loss during skin grafting procedures

Patients in the propranolol group underwent significantly larger skin grafting procedures (propranolol 4,500 ± 4,000 cm^2^ vs. control 3,300 ± 4,800 cm^2^; *P* = 0.01), yet required a similar number of packed red blood cell (PRBC) transfusions as the control group to maintain perioperative hematocrit levels (propranolol 4.4 ± 3.1 units vs. control 5.3 ± 5.4 units; *P* = 0.89). Average blood loss (estimated) was similar between groups (control 0.37 ± 0.73 ml/cm^2^ excised vs. propranolol 0.26 ± 0.21 ml/cm^2^ excised; *P* = 0.70; Table 2).

**Figure 5:**
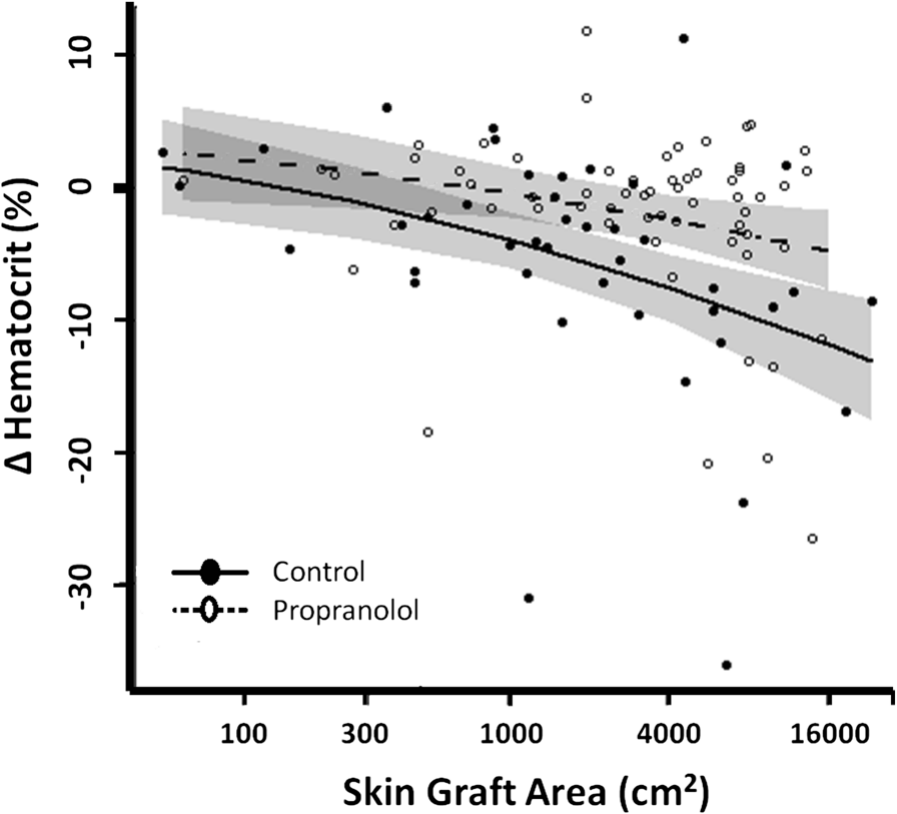
Propranolol significantly stabilizes perioperative hematocrit levels. Patients receiving propranolol maintained perioperative hematocrit levels compared to control patients. Propranolol was associated with a 1.6% improvement in perioperative hematocrit levels during grafting procedures with a graft area of 100 cm^2^, 2.5% improvement with 300 cm^2^, 3.6% improvement with 1,000 cm^2^, 5.2% improvement with 4,000 cm^2^, and 7.1% improvement with 16,000 cm^2^ (*P* = 0.002). Data are presented as adjusted mean ± 95% confidence intervals (shaded).

The relationship between skin graft area and change in perioperative hematocrit levels indicated that, as skin graft area increased, patients in the control cohort exhibited a significant decrease in perioperative hematocrit. In contrast, with increased skin graft area, patients on propranolol significantly maintained perioperative hematocrit levels (*P* = 0.002). A graft size of 4,000 cm^2^ was associated with a 5.2% improvement in perioperative hematocrit in the propranolol group over the control group (*P* = 0.002; Figure 5).

As expected, a correlation existed between mortality and graft size in all adults with burn injury, irrespective of treatment group. A significant difference was noted in both mean graft area per skin grafting procedure and in the sum of the areas used for all skin grafting procedures (Table 3).

**Table 3:** **Mortality and graft size**

**Parameter**	**Survivors (N = 52)**	**Nonsurvivors (N = 17)**	***P*** **value**
**Mean graft area**, cm^2^	3,440 ± 3,670	7,910 ± 3,420	**0.002**
**Total graft area**, cm^2^	9,960 ± 1,1150	20,170 ± 1,1340	**0.01**

### Fluid balance and acid base control

Net fluid balance was compared at 24, 48, and 72 hours post-admission. Twenty-four hours following hospital admission, net fluid balance was 11,100 ± 10,200 ml in the control cohort and 6,100 ± 5,800 ml in the propranolol cohort (*P* = 0.06). At 48 hours post-admission, net fluid balance was 6,500 ± 5,700 ml in the control cohort and 6,300 ± 7,500 ml in the propranolol cohort (*P* = 0.46). Finally, at 72 hours post-admission, net fluid balance was 3,900 ± 5,200 ml in the control cohort and 1,800 ± 2,800 ml in the propranolol cohort (*P* = 0.09). As these values did not significantly differ between groups, we cannot attribute hypotension to hypovolemia. Similarly, no significant difference in arterial pH was noted between groups over the first 72 hours following hospital admission. Twenty-four hours following admission, the pH was 7.23 ± 0.13 in control patients and 7.28 ± 0.08 in propranolol-treated patients (*P* = 0.08). At 48 hours post-admission, the pH was 7.32 ± 0.09 in control patients and 7.32 ± 0.10 in propranolol-treated patients (*P* = 0.85). Finally, 72 hours post-admission, the pH was 7.34 ± 0.15 in control patients and 7.39 ± 0.06 in propranolol-treated patients (*P* = 0.43). There were no significant differences between groups.

### Adverse events

Incidents of the following adverse events were recorded for patients in both groups: bradycardia (<60 beats/min), bradypnea (<12 breaths/min), hypotension (systolic <90 mm Hg, diastolic <60 mm Hg), and ischemia (mean arterial pressure <60 mm Hg). Adverse event incidence was comparable in each group. There were no significant differences between groups (Table 4).

**Table 4:** **Adverse events**

**Group**	**n (%)**	***P*** **value**
**Bradycardia**		
Control	3 (9)	0.47
Propranolol	6 (17)	
**Bradypnea**		
Control	13 (38)	0.63
Propranolol	16 (46)	
**Hypotension**		
Control	14 (41)	1.00
Propranolol	14 (40)	
**Ischemia**		
Control	13 (38)	0.23
Propranolol	19 (54)	

Bradycardia, pulse <60 bpm; bradypnea, respiratory rate <12 breaths/minute; hypotension, systolic/diastolic blood pressure <90/60 mm Hg; ischemia, mean arterial pressure <60 mm Hg.

## Discussion

One of the most significant contributors to decreased morbidity and mortality following severe burn injury has been the advent of early operative burn wound excision executed within the first 48 hours following burn injury [17-19]. Delayed excision has been associated with increased wound contamination, graft loss, hospital stay, hemorrhage, sepsis, and death [17-20]. Although early excision is associated with decreased hemorrhage, patients with severe burn injury often require multiple skin grafting procedures. Consequently, operative intervention is often required when intraoperative hemorrhage is at a peak, during 2 to 16 days postburn [17]. Therefore, pharmacotherapeutics that decrease intraoperative hemorrhage may augment hemodynamic recovery following surgical intervention in patients with burn injury.

In this prospective study, we investigated the effects of propranolol on the cardiovascular system and wound healing in 69 adults with severe burn injury. Demographic and burn injury characteristics did not differ between groups except with regard to TBSA burned. As patients in the control group sustained significantly larger burns over greater percentages of the TBSA, it is expected that these patients would exhibit more operative blood loss during burn excision. However, we have previously shown that, during primary burn excision in children, the area of TBSA burned is not a significant determinant of blood loss. Rather, the area of devitalized tissue excised contributes to approximately 50% of the variability in blood loss during skin grafting procedures. Furthermore, the extent of full-thickness burn injury is associated with significantly more blood loss as well [21]. Therefore, the implementation of a bias due to the significant difference in TBSA burned between groups in this study was not of concern as both groups experienced a similar severity of full-thickness burn (control 45 ± 29% vs. propranolol 40 ± 22%; *P* = 0.48).

Propranolol significantly reduced the time between grafting surgeries by one week, on average. This data is consistent with results from a study of 79 Iranian patients with moderate burn injury (approximately 30% TBSA burns) in which propranolol improved healing times of both partial and full-thickness burns [22]. However, it should be noted that the investigators of the aforementioned study excised burn wounds according to a delayed approach, approximately one month after initial burn injury.

β-adrenergic receptors are found widely in cutaneous fibroblasts, keratinocytes, and endothelial cells. Pullar and colleagues reviewed the effects of β_2_-adrenergic receptor modulation on wound repair and reported modulatory effects on cell proliferation and migration via galvanotaxis, inflammation via neutrophil chemotaxis, wound contraction and re-epithelialization via keratinocyte migration and fibroblast mediation, and angiogenesis via cyclic AMP-mediated vascular endothelial-derived growth factor expression [23]. In fact, β_2_-adrenergic receptor inhibition in a murine model resulted in increased dermal fibroblast function and re-epithelialization during the early stages of wound repair [24]. Alterations in microvascular blood flow induced by β-blockade may play a role in wound healing as well. In a rodent model, low (2 mg/kg) and high (20 mg/kg) doses of propranolol resulted in 35% lower cortical regional cerebral blood flow than the control treatment. Additionally, propranolol significantly reduced mean oxygen consumption without effecting oxygen saturation levels compared to the control treatment [25]. Regarding coagulation, propranolol abolished isoproterenol-induced increases in plasma von Willebrand factor antigen levels but had no effect on tissue factor or D-dimer expression in hypertensive patients. These data suggest that propranolol may protect against endothelial cell damage, as increased von Willebrand factor levels are indicative of vascular injury [26]. Here we report significantly faster donor site wound healing times in patients receiving propranolol following burn injury. Ongoing studies of the samples from these patients will allow for elucidation of the molecular effects of propranolol that underlie improved healing after burn injury.

On average, patients in the propranolol cohort underwent operative procedures with graft areas 1,200 cm^2^ larger than those in the control group. The goals of early excision of the burn wound include the removal of all devitalized and necrotic tissue, which if left untreated, would provoke bacterial contamination and impede adequate wound healing. More often than not, an accurate assessment of partial thickness and full-thickness burns cannot be obtained until patients undergo operative intervention. Burn patients are assessed on a case-by-case basis with final evaluation of skin graft area being decided within the operating theater. Error in primary clinical assessment of viable and nonviable tissue likely explains the difference in final graft area required. As estimated blood loss is often an inaccurate and subjective assessment of hemorrhage, we decided to measure blood loss using an objective approach. Perioperative hematocrit levels were plotted against skin graft area, accounting for severity of injury and surgical intervention for each patient. We found that propranolol administration was associated with a 5% improvement in perioperative hematocrit levels during skin grafting procedures of 4,000 cm^2^. Moreover, the attenuation of blood loss was even more profound after larger skin grafting procedures. These findings may be attributed to the various effects of propranolol on the cardiovascular system.

Direct inhibition of β-adrenergic receptors in the heart decreases heart rate, blood flow, and cardiac output. Angiogenesis may be attenuated via vasoconstriction, and decreased expression of matrix metalloproteinases, basic fibroblast growth factor, and vascular endothelial growth factor may limit operative hemorrhage with propranolol administration. Similarly, propranolol enhances upregulation of capillary endothelial cells and apoptosis, further limiting angiogenesis and blood loss [11,27]. Finally, earlier work by our group has provided evidence that propranolol administration significantly reduces peripheral perfusion via increased leg vascular resistance in adults with severe burn injury [7]. However, in this work, we observed a reduction in arterial and venous lactate levels in both nonburned and burned adults following two hours of intravenous propranolol administration. β-blockade was thought to have reduced lactate production via inhibition of lipolysis [7]. In the current study, we found no difference in arterial pH between groups over the first 72 hours following hospital admission. Therefore, decreased chronotropic effects during early resuscitation in patients treated with propranolol did enhance acidosis further than expected by the nature of severe burn injury, as evidenced in the control group.

Resuscitative efforts with either crystalloid or colloid fluids have long been a subject of debate. One of the limitations of the current study was the omission of comprehensive resuscitation data in our analysis. However, administration of PRBC is likely the strongest contributor to altered perioperative hematocrit levels in adults with burn injury. As no difference in PRBC administration was noted between groups and target hemoglobin levels remained constant among groups, we are confident that resuscitation with various other colloid and crystalloid products follow a similar pattern.

Finally, it bears mentioning that the findings from our study may not be limited to burn injury. For example, propranolol may attenuate hemorrhage perioperatively in patients on anticoagulant therapy requiring emergency surgical intervention. Alternatively, propranolol may improve wound healing times after bedside or surgical debridement for various diseases. The effect of propranolol on wound healing and hemorrhage in other injury/disease models deserves further investigation.

## Conclusions

In severely burned adults, administration of propranolol during acute hospitalization diminishes blood loss during skin grafting procedures and speeds wound healing.

## Key messages

- Propranolol, given throughout hospitalization at a dose that reduces admission heart rate by 20%, speeds wound healing by one week, on average.
- Propranolol administration also diminishes blood loss, as assessed by perioperative hematocrit levels. This effect becomes more profound as skin graft area increases.

## Abbreviations

LOS: length of stay
PRBC: packed red blood cells
TBSA: total body surface area
